# Effects of Co-Contamination of Microplastics and Cd on Plant Growth and Cd Accumulation

**DOI:** 10.3390/toxics8020036

**Published:** 2020-05-20

**Authors:** Fayuan Wang, Xiaoqing Zhang, Shuqi Zhang, Shuwu Zhang, Catharine A. Adams, Yuhuan Sun

**Affiliations:** 1 College of Environment and Safety Engineering, Qingdao University of Science and Technology, Qingdao 266042, China; wangfayuan@qust.edu.cn (F.W.); zhxqing2020@163.com (X.Z.); zsq9629@163.com (S.Z.); zhangshuwu@126.com (S.Z.); 2 Shandong Key Laboratory of Water Pollution Control and Resource Reuse, School of Environmental Science and Engineering, Shandong University, Qingdao 266237, China; 3 Department of Plant and Microbial Biology, University of California Berkeley, Berkeley, CA 94704, USA; catadams@berkeley.edu

**Keywords:** heavy metals, soil contamination, environmental risk, cadmium, phytoavailability

## Abstract

Microplastics (MPs) occur widely in terrestrial ecosystems. However, information on the interaction of MPs with metals in terrestrial ecosystems is lacking in the literature. The present study investigated the effects of two types of MPs (high-density polyethylene (HDPE) and polystyrene (PS)) with different dosages (i.e., 0, 0.1%, 1%, and 10%) on the uptake and effects of Cd in maize plants grown in an agricultural soil. Results showed that addition of Cd at a 5 mg/kg caused inhibited plant growth and resulted in high Cd accumulation in plant tissues. Polyethylene alone showed no significant phytotoxic effects, but a high-dose of HDPE (10%) amplified Cd phytotoxicity. Polystyrene negatively affected maize growth and phytoxicity further increased in the presence of Cd. Both HDPE and PS caused soil diethylenetriaminepentaacetic acid (DTPA)-extractable Cd concentrations to increase but did not significantly affect Cd uptake into plant tissues. In the soil without Cd addition, HDPE decreased soil pH, while PS did not significantly alter soil pH. However, in the soil spiked with Cd, both HDPE and PS increased pH. Overall, impacts on plant growth and Cd accumulation varied with MP type and dose, and PS induced substantial phytotoxicity. In conclusion, co-occurring MPs can change Cd bioavailability, plant performance, and soil traits. Our findings highlight the ecological impacts that could occur from the release of MPs into soil.

## 1. Introduction

Microplastics (MPs), which are defined as plastic debris with a size smaller than 5 mm, are considered emerging contaminants and are attracting global concern [1,2,3]. Recently, the occurrence and effects of MPs in terrestrial ecosystems such as agricultural soils have attracted particular interest [4,5,6]. Microplastics can enter soils through various pathways, such as the application of biosolids [4,7], organic fertilizers [8], and the use of plastic mulch films [9]. A series of studies have confirmed the occurrence of MPs in agricultural soils [9,10,11]. The occurrence of MPs in soils may effect soil-dwelling microbes, animals, and plants, posing potential threats to agroecosystems [9,11,12]. Microplastics can alter soil structure and properties and plant performance, thus disrupting the resident microbial community [6,9,11]. However, research gaps exist on the ecological impacts and toxicity of MPs in agroecosystems. 

Metals and metalloids are common contaminants in farmlands [13,14]. For instance, Cd ranks as the most common metal contaminant of farmland soils in China [15]. Due to their wide co-occurrence, MPs and Cd could interact with each other to affect the bioavailability and toxicity of the metal in agroecosystems [16]. In particular, MPs can adsorb metallic contaminants and thus serve as a vector for carrying the metals into organisms [17,18]. Our previous studies found that Cd adsorbed onto high-density polyethylene (HDPE) MPs was easily desorbed [18], and the presence of HDPE MPs decreased Cd adsorption but increased desorption of Cd by soil [19]. Nevertheless, how MPs influence the biological effects of heavy metals such as Cd in plant–soil systems remains unknown [16]. 

Given the above concerns, we speculate that the co-occurrence of MPs and Cd can change the bioavailability and toxicity of Cd, with consequent impacts on plant growth and Cd accumulation. Here, a soil microcosm experiment was conducted to test the effects of two common MPs (high-density polyethylene and polystyrene) on the uptake and effects of Cd accumulation in maize grown in an agricultural soil. The present study explores the interactions between MPs and toxic metals in a soil–plant system. 

## 2. Materials and Methods

### 2.1. Soil, Plant, and MPs 

The soil for plant culture was collected from farmland located at Nanzhangyuan Village, Jimo District, Qingdao, China. Fresh soil was air-dried after removing animal and plant residues, stones, and other debris. The air-dried soil was sieved using a 2 mm sieve for analysis and pot culture. The detailed properties of the soil are shown in Table 1. 

Two types of common MPs, high-density polyethylene (HDPE) and polystyrene (PS), were selected as the study MPs. The HDPE was purchased from Dongming Plastic Material Co. Ltd, Guangdong, China. The HDPE has a density of 0.940–0.976 g/cm^3^, a degree of crystallinity of approximately 80%–90%, and a softening point of 125–135 °C. General-purpose PS (PS121) was purchased from TRINSEO (Styron), with a density of 1.05 g/cm^3^, a melting point of 240 °C, and a glass transition temperature of 80–105 °C. The MPs were sieved manually to ensure a particle size of 100–154 μm. Prior to use, MPs were cleaned using 0.1 mol/L HCl to remove potential metals on their surface. 

Seeds of maize (*Zea mays* L. var. Wannuoyihao) were purchased from Hebei Huasui Seed Industry Co., Ltd, China. The seeds were surface-sterilized with 2% NaClO solution for 15 min and subsequently washed several times with distilled water. 

### 2.2. Experimental Set-Up and Procedure

We designed a trifactorial experiment including (1) two types of MPs, i.e., HDPE and PS; (2) four doses of MPs, i.e., 0%, 0.1%, 1%, and 10% (*w*/*w*); and (3) two Cd concentrations, i.e., 0 and 5 mg Cd /kg soil. Thus, a total of 14 treatments were applied, each treatment with four replicates. The doses of MPs were designed based on the current knowledge of MP occurrence in soils [5,7] and previous studies [6,20]. The Cd concentration (5 mg/kg) represents an environmentally realistic level reported in China’s farmland soil [21]. 

The MPs were thoroughly mixed into the soil to achieve the target doses. The Cd solution was prepared by dissolving Cd(NO_3_)_2_·4H_2_O in distilled water and then was spiked into the soil–MP mixture to obtain the target concentration. The soil samples were equilibrated at room temperature for one week prior to sowing seeds. Twenty uniform surface-sterilized seeds were grown in each pot (height 11 cm, diameter 18 cm) with 880 g of air-dried soil-MP mixture. After seedling emergence, ten seedlings of uniform size were retained in each pot. All pots were randomly placed in a plant growth chamber with a day/night (12/12 h) temperature regime of 25–28/20–23 °C, a light intensity of 10,000 Lux, and a relative humidity of 50%–55%. During the plant growth period, distilled water was irrigated every other day to maintain soil water content between 12% and 18%. The plants were harvested for analysis one month after seedling emergence. The soil from each pot was mixed thoroughly, and 50 g of soil sample was taken for analysis of soil pH and available Cd. 

### 2.3. Plant and Soil Analysis

After cleaning with tap water and deionized water, the fresh weights of the shoots and roots were each measured. The dry weights were determined after oven-drying the fresh materials at 70 °C for 24 h. 

The dried plant material was ground and then wet-digested using HNO_3_. The Cd concentration in the digested solution was estimated with an inductively coupled plasma mass spectrometer (ICP-MS, iCAP RQ, Thermo Fisher Scientific Inc., Waltham, MA, USA). Soil-available Cd was extracted using diethylenetriaminepentaacetic acid (DTPA) solution (0.005 M DTPA, 0.1 M triethanolamine, 0.01 M CaCl_2_, pH 7.3) based on the method described previously [22]. The suspension (soil:DTPA solution ratio, 1:2, *w*/*v*) was shaken at 180 rpm and 25 °C for 2 h and then filtered with filter paper and a Millipore 45 μM filter. The Cd concentration was determined with an atomic absorption spectrophotometer (FAAS, AA-7000, Shimadzu, Kyoto, Japan), based on Chinese standard GB/T 23739-2009 “Soil quality—Analysis of available Lead and Cadmium contents in soils: Atomic absorption spectrometry”. Cd standard solutions with concentrations of 0, 0.5, 1.0, 1.5, 2.0, and 2.5 mg/L were used. For quality control of metal analysis, guaranteed-grade reagents and double-deionized water were used during the analysis procedure. Three blanks were included. The recovery rate was estimated to 96% ± 3%. The pH of soil suspensions (soil:water ratio, 1:2.5, *w*/*v*) was measured using a pH meter (pHS-3C, Sanxin, Shanghai, China). To ensure quality assurance and quality control of the data, reagent blanks and standard solutions were included during sample analysis. Bioaccumulation index (BAI) was calculated as the ratio of Cd concentrations in plant tissues and Cd concentrations in soil [23]. 

### 2.4. Statistical Analysis

Data analysis was performed using SPSS 23.0. All figures were generated using Excel 2013. Kolmogorov-Smirnov test was performed to evaluate the normality of data. Significance among treatments was then compared using Duncan’s multiple range test at *p* < 0.05. Two-way and three-way ANOVA analyses were conducted to analyze the significance of interactions among MP type, MP dose, and Cd. Pearson correlation coefficients were calculated to analyze the correlation among the different parameters. 

## 3. Results and Discussion

### 3.1. Plant Biomass

**Figure 1:**
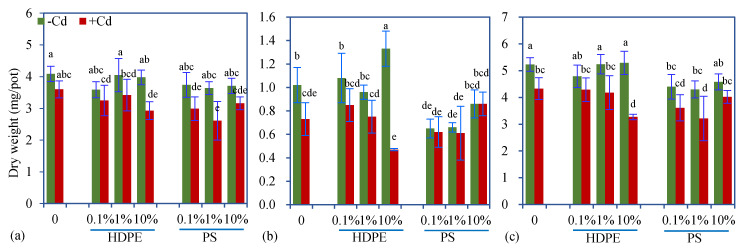
Shoot (**a**), root (**b**) and total (**c**) dry weights (mean ± SE, *n* = 4) of maize plants exposed to microplastics (MPs) with or without Cd. +Cd and -Cd represent the treatments with or without 5 mg/kg Cd, respectively. Different letters over the bars indicate significant differences using a one-way ANOVA followed by Duncan’s multiple range test (*p* < 0.05). Two-way and three-way ANOVA results are shown in Table 2 and Table 3, respectively.

In most cases, PS decreased shoot and/or root dry weights, resulting in lower total dry weights, but the effects varied with Cd (Figure 1). In the presence of Cd, the inhibitory effect of PS on growth was further increased. HDPE did not significantly decrease plant biomass and even exhibited stimulatory effects on root growth when applied alone at the 10% dose. However, when applied jointly with Cd, HDPE at the dose of 10% decreased plant dry weights. Two-way and three-way ANOVA results showed that the phytotoxicity of MPs was highly dependent on MP type and dose (Table 2 and Table 3). Polyethylene showed no obvious phytotoxic effects, while PS exhibited significant phytotoxicity even at a low dose (0.1%). 

Thus far, the effects of MPs on terrestrial plants are unknown, particularly under soil culture conditions [11]. The mechanisms by which MPs exert toxicity in plants have been explored using solution culture experiments. In a 72 h seed germination bioassay, MPs were found to accumulate in the pores of seed capsules of *Lepidium sativum* [24], suggesting that physical blocking of water uptake may account for the delayed seed germination and root growth. In our previous sand culture experiment, HDPE MPs with a size of 23–38 μm and at a dose of 100 mg/g inhibited water absorption and growth of mung bean [25]. In another experiment, 100 nm PS MPs (10, 50, and 100 mg/L) caused both genotoxic and oxidative stress in *Vicia faba* plants [26]. In particular, 100 nm PS can accumulate in the *V. faba* root [26], and 200 nm PS can enter lettuce roots and then be transferred to the stems and leaves [27], thus causing potential damage to subcellular structures. Mechanisms such as these may partially explain the phytotoxicity of PS in maize observed in the present study. 

A few studies have been conducted in soil–plant systems [6,20,28,29], and the results showed the effects of MPs on plants were quite conflicting, ranging from negative [20,28] to neutral [29] and to positive [6]. Our results for HDPE are similar to those of previous studies that found slight growth effects of low-density PE (50 μm–1 mm, 1%) and HDPE (2–3 mm, 2%) on wheat [20] and spring onions [6], respectively. In the present study, HDPE of a smaller size (100–154 μm) and higher dose (10%) still showed no inhibition and even stimulated root biomass when applied alone. However, PS at the doses of 0.1%–10% generally decreased plant biomass, which completely contradicts previous findings that demonstrated that PS (2–3 mm, 2%) exerted plant growth and promoted effects on spring onion [6]. In other studies, macrosized expanded PS (8.3 ± 0.5 mm) caused no adverse impacts on three crops (mung bean, lettuce, and rice) [30]. These differences in plant impact suggest that particle size may also be a determining factor influencing the phytotoxicity of PS. Microplastics with a similar shape and size to those of soil particles may cause smaller soil and plant responses [6]. Thus, the same MPs may have varied impacts on plants growing in different soils. By integrating the current findings, it can be concluded that the phytotoxicity of MPs is dependent on plastic type, particle size, and exposure dose. 

The present study found that Cd caused toxic effects on maize. Excess Cd can induce a series of negative effects on morphological, physiological, and biochemical functions of plants via various mechanisms, such as interfering with nutrient uptake, inducing oxidative stress, and causing genotoxicity [31]. Here, Cd(NO_3_)_2_ was used to simulate Cd contamination in soil. Cd(NO_3_)_2_ is a salt with high solubility and bioavailability, which may account for its significant phytotoxicity. Under field conditions, Cd may not occur as a soluble salt and probably has a lower toxicity. 

More intriguingly, the growth inhibition induced by Cd varied with MP type and dose (Table 2 and Table 3). Polystyrene had no significant interaction with Cd (two-way ANOVA results, Table 2), but HDPE at the dose of 10% amplified the negative effect of Cd on root biomass (Figure 1b). In combination with 10% HDPE, Cd produced the most significant inhibition on shoot and root dry weights (Figure 1), suggesting that the co-occurrence of HDPE has more significant influences on Cd phytotoxicity than PS. The HDPE and PS used consisted of different plastic monomers, which determine their structures and properties. For example, PE is classified as a rubbery plastic, but PS is considered a glass plastic, and PE has a lower density than PS. Microplastics of different plastic types vary with their affinity and adsorption capacity for chemicals [32]. Therefore, they are likely to interact with Cd to produce various effects on plant performance, which continues to be discussed in the following subsections. 

### 3.2. Cd Concentration and Uptake in Plants

Cd was not detected in the plants that received no Cd, but high Cd concentrations were observed in both shoots and roots of maize plants exposed to Cd (Table 4). Compared to the control, both HDPE and PS did not significantly change Cd concentrations and uptake in shoots or roots (Table 4). Two-way ANOVA results showed significant interactive effects on root Cd concentration between HDPE and Cd (Table 2). 

To date, no research has been conducted on the impacts of MPs on heavy metal accumulation in plants. Only two studies have been conducted using earthworms and MPs: One found the addition of HDPE MPs in soil had no significant effects on Zn accumulation, mortality, and weight change of earthworms [17]; the other showed the presence of polyvinyl chloride MPs lessened the accumulation of total As and the reduction of As(V) in earthworms [33]. These findings indicate a combined toxicity between MPs and heavy metal(loid)s in soil.

Cd generally has a high BAI in plant tissues due to its low adsorption coefficient and high soil–plant mobility [31]. In the present study, due to the low Cd background value of the selected soil, Cd was not detected in either shoots or roots of maize plants which were grown in soil without additional Cd, irrespective of MPs (Table 4). In the soil spiked with Cd, plants accumulated a high amount of Cd in both shoots and roots, with a BAI of 3.73–4.34 in shoots and 6.66–9.29 in roots, respectively (Table S1), confirming that the Cd added to soil is highly bioavailable and mobile in the soil–plant system. 

Cd accumulation by plants is dependent on soil Cd content and availability, soil characteristics, and plant traits [31], which can all be influenced by MPs. First, MPs can decrease the Cd adsorption capacity of soil [19]. The co-occurring MPs may increase Cd bioavailability and Cd uptake by plants. Second, due to their hydrophobic surfaces, MPs may interfere with the absorption by roots of water and Cd from soil solution. In fact, MPs have been shown to alter water evaporation and water availability in soil and to increase the availability of nutrients [6]. Microplastics can also cause shifts in soil biophysical properties, such as soil bulk density and aggregation [34], which may consequently influence Cd bioavailability and bioaccumulation. Putatively, the presence of MPs may inhibit plant uptake of water and Cd. Third, MPs influence root traits such as root biomass, root length, and tissue density [6], thereby changing root uptake of Cd. We found the addition of PS increased soil-available Cd but decreased root biomass, which can partially explain the nonsignificant increase in plant Cd accumulation. In sum, MPs can produce positive, neutral or negative impacts on Cd, soil, and plants. The nonsignificant effects of MPs on plant Cd accumulation may be a “compromise” of these various functions.

### 3.3. DTPA-Extractable Cd in Soil

DTPA-extractable Cd concentrations were not detected in soil that received no Cd (Table 4). When 5 mg/kg Cd was added into the soil, DTPA-extractable Cd concentrations were increased by PS, followed by HDPE, except for the 10% dose. Two-way and three-way ANOVA results confirmed that the effects of MPS on DTPA-extractable Cd varied depending on MP type and dose (Table 2 and Table 3). 

Due to their relatively simple components and surface structure compared to soil constituents, MPs generally have a highly hydrophobic surface with a much lower adsorption capacity for heavy metals [17,19]. Our previous study found that HDPE MPs reduced soil adsorption for Cd but elevated the desorption rate of the adsorbed Cd [19], implying that co-occurring MPs may enhance the release of exchangeable Cd from sorption sites. Thus, the presence of MPs may increase Cd exchangeability and attenuate soil retention of Cd via a “dilution effect”. Furthermore, the surface of MPs can be electrostatically charged [35]. Thus, MPs can interact with soil constituents (e.g., organic matter), hence occupying the sorption sites on these constituents and hindering the adsorption of Cd. 

However, with an increasing MP dose, the concentration of DTPA-extractable Cd did not increase but even decreased, and HDPE at the dose of 10% showed a slight but significant reduction (Table 4). These mixed results imply the impacts of MPs on Cd extractability in soil–MP mixture are complex. In addition to their direct interaction with Cd, MPs may change soil adsorption capacity for Cd via changing soil structure and properties [6]. In the presence of soil biota, polyester microfiber can reduce soil aggregate stability [36], which may further decrease the exchangeability of Cd bound to soil particles. Furthermore, MPs may indirectly influence forms of Cd via modification of root exudates and rhizospheric traits. One line of evidence that MPs indirectly influence Cd is that high-dose HDPE induced higher soil pH (see Section 3.4), which can partially explain the lower DTPA–Cd. Another piece of evidence is that HDPE at the 10% dose produced a plant-growth-stimulating effect when applied alone (Figure 1), but an inhibition when applied jointly with Cd. This implies HDPE may change plant performance to cope with Cd, thereby altering Cd forms and bioavailability. 

Considering the different impacts of HDPE and PS, plastic traits other than particle size and dose can have a greater effect on increasing DTPA-extractability of Cd. Plastic type affects the sorption characteristics of chemicals by MPs: Rubbery plastics and low-density plastics generally have a stronger affinity and a higher adsorption capacity for chemicals than glassy and high-density plastics [32]. Polyethylene is classified as a rubbery plastic with lower density than glassy PS. In the current experiment, PS caused a greater increase in DTPA-extractable Cd than HDPE, which can be attributed to their different plastic properties. For example, due to its higher density than HDPE, PS of equal mass has a smaller volume than HDPE, and possibly a lower Cd capacity. Thus, PS probably has a stronger “dilution effect” than HDPE. Moreover, MPs of different plastic types cause various changes in soil properties and plant performance [6,34], which may indirectly alter Cd behavior and availability in soil. 

### 3.4. Soil pH

**Figure 2:**
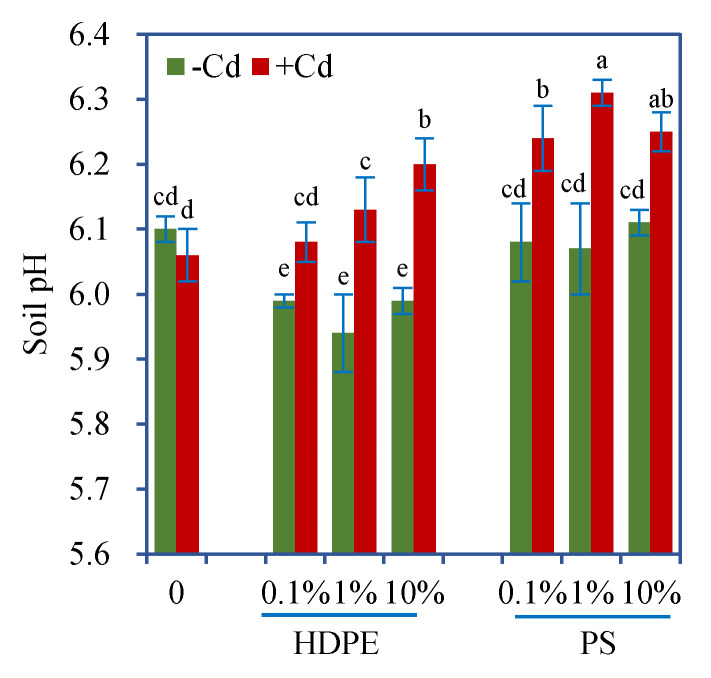
pH (mean ± SE, *n* = 4) of soil exposed to MPs with or without Cd. +Cd and -Cd represent the treatments with or without 5 mg/kg Cd, respectively. Different letters over the bars indicate significant differences using a one-way ANOVA followed by Duncan’s multiple range test (*p* < 0.05). Two-way and three-way ANOVA results are shown in Table 2 and Table 3, respectively.

Cd alone caused a slight but nonsignificant decrease in soil pH (Figure 2). However, when in the presence of MPs, addition of Cd generally increased soil pH, and the effects varied with MP type and dose (Figure 2). Polyethylene alone generally decreased soil pH, whereas PS alone showed no significant influence. When in the presence of Cd, both MPs increased soil pH, and PS produced a more pronounced effect. Two-way ANOVA results showed that both HDPE and PS had signifciant interactive effects with Cd on soil pH (Table 2).

To date, little is known regarding the impacts of MPs on soil pH. A recent study by Boots et al. [37] found that soil pH decreased significantly after 30 days of exposure to HDPE MPs (0.1%, *w*/*w*), which could be attributed to altered cation exchange in the soil by the presence of HDPE particles. Similarly, we found a lower pH in the soil exposed to HDPE alone. However, another study showed that after two-months of soil culture, low-density PE with a size of 50 μm to 1 mm induced an increase in soil pH, which further increased after another two-month culture [28], but the underlying mechanisms causing this increase remain unclear. These findings suggest the need for further investigations on how MPs influence soil pH. Although the two MPs are both nonbiodegradable, they have different plastic components and properties and hence may cause different effects on soil properties and plant traits [6,34], as well as soil on microbial community and acitivity [6,38,39,40]. Furthermore, the effects of MPs are dependent on experimental conditions. The impacts of PE on richness and diversity of the bacterial communities can vary ranging from negative [40] to insignificant [39] and to positive [41]. Hence, all these changes may ultimately influence soil pH. More interestingly, both MPs, especially PS, increased the pH of soil spiked with Cd, confirming the interactive effects between MPs and Cd on soil pH. The possible reason for this pH increase may be ascribed to the changes in plant and microbial metabolites induced by MPs and Cd, as they both can cause oxidative damages in plants and soil biota [11,26,42]. 

Soil pH is a key factor determining Cd mobility, bioavailability, and phytoaccumulation, which are generally higher at the lower soil pH [31,43]. Unexpectedly, the co-occurrence of HDPE and PS induced higher soil pH and higher DTPA-extractable-Cd concentrations. This finding is contrary to much previously published research. In addition to pH, many other soil characteristics such as organic matter and soil microbial activity can also affect Cd bioavailability [31]. In a complex soil–plant system, the presence of MPs may bring complicated impacts on soil properties, not merely in terms of pH, which could collectively affect Cd availability. Accordingly, the changes in soil pH can be considered a result of multiple factors, but not the cause of varied availability of Cd.

### 3.5. Correlation Analysis 

Pearson correlation analyses showed a positive correlation between DTPA-extractable Cd and the Cd added in soil, both of which correlated negatively with plant dry weights and positively with plant Cd concentrations (Table 5). These findings are in accordance with previous conclusions [31]. In the present study, although MPs caused no significant changes in Cd concentrations and total uptake in plants, the presence of MPs, particularly PS, substantially increased Cd bioavailability in soil (Table 4), implying an additional risk to soil–plant systems. In particular, MPs may also change the bioavailability and plant uptake of nutrients with chemical behaviors similar to Cd and then alter the quality and nutritional value of crops. Given the present microcosm experiment, lifecycle experiments should be conducted to elucidate plant growth and Cd accumulation as influenced by MPs.

### 3.6. General Discussion

The present results clearly show some negative impacts of MPs on plant growth as well as environmental risks of Cd, with implications for agricultural applications of plastic products. For example, agricultural mulch film consumption reached up to 1.455 million tons in China in 2015 [44]. However, plastic film residues after crop harvest are generally difficult to remove or recycle from the field, ultimately leading to MP contamination. Thus, policy makers should take measures to reduce soil contamination of MPs, particularly in agricultural fields, such as encouraging the use of biodegradable plastics, improving recovery rate of plastic residues, and remediating MP-contaminated soils.

The large knowledge gaps on co-contamination of MPs and contaminants in soil provide many avenues for further research. First, the current studies, including the present results, were mainly based on short-term microcosms under laboratory conditions. In the future, long-term and large-scale studies should be performed on the interactions of MPs and heavy metals in agroecosystems, particularly in food crops, to ensure food safety. Second, because the impacts of MPs usually vary greatly with plastic type, particle size and shape, and surface characteristics [19,20,41,45,46], further research should focus on the ecotoxicity of more MPs, particularly nanoscale plastics and aged MPs, and their associated contaminants. Third, PS produced stronger impacts on Cd bioavailability and plant growth than HDPE. Due to their differences in polymer type and components, PS and HDPE may have different concomitant components. Consequently, in addition to the impacts of MPs particles, the release of concomitant components during the degradation process, such as additives, plasticizers, and minerals, may also cause uncertain effects on soil and plants [47,48], which should be taken into consideration, especially in long-term experiments. Lastly, the biotic response to contaminants varies not only with contaminant properties, but also with the organisms and soil. As such, comparative studies using more combinations of MPs and contaminants, as well as plant species, are needed to fully recognize the behavior, fate, bioavailability, and toxicity of co-occurring MPs and toxic metals in agroecosystems. 

## 4. Conclusions

For the first time, we studied the effects of MPs on plant growth and Cd accumulation in Cd-contaminated soil. When applied alone, HDPE showed no negative effects on maize plants, while PS produced significant phytotoxicity. Both HDPE and PS increased DTPA-extractable-Cd contents in soil but did not alter Cd accumulation in plant tissues. Compared to HDPE, PS produced more pronounced effects on Cd bioavailability and plant growth inhibition, implying its higher risk in soil–plant systems. Although HDPE alone had no phytotoxicity, high-dose HDPE amplified the negative effect of Cd on root biomass. Thus, co-occurring MPs may change Cd availability and plant performance. Given the current knowledge gap, long-term and large-scale experiments should be conducted using various MPs and contaminants co-occurring in real environments to unveil their impacts on soil ecosystems. 

## Figures and Tables

**Table 1:** Physical and chemical properties of the test soil.

pH		6.0
Available P (mg/kg)		153.6
Available K (mg/kg)		178.0
Nitrate N (mg/kg)		20.5
Total N (g/kg)		0.816
Total Cd (mg/kg)		0.16
Cation exchange capacity (cmol/kg)		7.53
Organic matter (g/kg)		13.1
Soil type		Alfisols (US soil taxonomy)
Soil texture		Sandy loam
Soil particles distribution		
	2.0~0.05 mm (%)	68.6
	0.05~0.002 mm (%)	22.9
	<0.002 mm (%)	8.5

Note: The total Cd was determined using graphite furnace atomic absorption spectrophotometry after digesting with HCl-HNO_3_-HF-HClO_4_.

**Table 2:** Effects of MPs, Cd, and their interactions on measured variables based on a two-way ANOVA analysis. Significance level (*F* value): * *p* < 0.05, ** *p* < 0.01, *** *p* < 0.001; ns: nonsignificant effect.

Variables	HDPE			PS		
	HDPE dose	Cd	HDPE × Cd	PS dose	Cd	PS × Cd
Shoot dry weight	2.09 ns	17.66 ***	1.24 ns	6.00 **	32.98 ***	0.96 ns
Root dry weight	0.51 ns	36.87 ***	5.35 **	7.79 **	3.79 ns	1.74 ns
Total weight	1.76 ns	45.15 ***	3.53 *	6.88 **	25.54 ***	0.46 ns
Soil pH	3.48 *	64.86 ***	15.15 ***	10.03 ***	63.82 ***	12.85 ***
DTPA-Cd	42.61 ***	29324.66 ***	42.61 ***	16.97 ***	11577.01 ***	16.97 ***
Shoot Cd conc.	0.19 ns	326.88 ***	0.19 ns	0.88 ns	743.91 ***	0.88 ns
Root Cd conc.	7.89 **	1861.31 ***	7.89 **	1.21 ns	665.48 ***	1.21 ns
Shoot Cd uptake	1.00 ns	422.60 ***	1.00 ns	5.12 **	1955.10 ***	5.12 **
Root Cd uptake	0.98 ns	144.64 ***	0.98 ns	3.02 *	228.05 ***	3.02 *

**Table 3:** Effects of MP type, MP dose, Cd, and their interactions on measured variables based on a three-way ANOVA analysis. Significance level (*F* value): * *p* < 0.05, ** *p* < 0.01, *** *p* < 0.001; ns: nonsignificant effect.

Variables	Type (T)	Dose (D)	Cd	T × D	T × Cd	D × Cd	T × D × Cd
Shoot dry weight	4.54 *	0.08 ns	41.66 ***	3.25 *	0.25 ns	0.69 ns	1.98 ns
Root dry weight	18.59 ***	3.02 ns	30.33 ***	3.28 *	19.47 ***	4.69 *	6.05 **
Total dry weight	11.85 ***	0.07 ns	48.56 ***	3.57 *	1.66 ns	1.61 ns	3.19 *
Soil pH	101.51 ***	4.01 *	97.59 ***	2.64 ns	0.45 ns	4.74 *	2.97 ns
DTPA-Cd	41.48 ***	33.96 ***	22877.90 ***	2.11 ns	41.48 ***	33.96 ***	2.11 ns
Shoot Cd conc.	0.05 ns	0.76 ns	663.15 ***	0.09 ns	0.05 ns	0.76 ns	0.09 ns
Root Cd conc.	1.99 ns	4.73 *	1380.44 ***	1.24 ns	1.99 ns	4.73 *	1.24 ns
Shoot Cd uptake	2.84 ns	1.55 ns	943.67 ***	1.35 ns	2.84 ns	1.55 ns	1.35 ns
Root Cd uptake	0.59 ns	1.47 ns	274.506 ***	3.67 *	0.59 ns	1.47 ns	3.67 *

**Table 4:** Diethylenetriaminepentaacetic acid (DTPA)-extractable Cd concentrations in soil, and Cd concentrations and uptake in maize plants exposed to MPs with Cd. Different letters following the mean values ± SD (*n* = 4) in the same column indicate significant differences using a one-way ANOVA followed by Duncan’s multiple range test (*p* < 0.05). Two-way and three-way ANOVA results are shown in Table 2 and Table 3, respectively.

MP Treatment		DTPA-Cd (mg/kg)	Cd conc. in Plants (mg/kg)		Cd Uptake in Plants (mg/pot)	
Type	Dose	Soil	Shoots	Roots	Shoots	Roots
Control	0	3.46 ± 0.04c	20.15 ± 2.40a	39.30 ± 2.83ab	0.072 ± 0.005a	0.029 ± 0.007ab
HDPE	0.1%	3.82 ± 0.11b	22.23 ± 1.87a	46.18 ± 6.53a	0.072 ± 0.011a	0.035 ± 0.015ab
	1%	3.81 ± 0.07b	21.84 ± 7.94a	34.34 ± 1.57b	0.073 ± 0.023a	0.026 ± 0.005b
	10%	3.26 ± 0.09d	20.44 ± 3.92a	44.43 ± 2.21a	0.059 ± 0.007a	0.025 ± 0.008b
PS	0.1%	4.08 ± 0.15a	21.75 ± 3.97a	44.86 ± 8.66a	0.064 ± 0.007a	0.028 ± 0.008ab
	1%	4.05 ± 0.14a	22.38 ± 3.79a	42.08 ± 7.49ab	0.057 ± 0.007a	0.025 ± 0.010b
	10%	3.73 ± 0.19b	19.25 ± 1.26a	47.96 ± 6.58a	0.061 ± 0.004a	0.041 ± 0.008a

**Table 5:** Pearson correlation coefficient (*r*) between soil and plant parameters. Significant level: * *p* < 0.05, ** *p* < 0.01, *** *p* < 0.001; ns: nonsignificant effect.

*r*	Shoot Dry Weight	Root Dry Weight	Total Dry Weight	DTPA-Cd	Shoot Cd conc.	Root Cd conc.
Cd added in soil	−0.646 ***	−0.432 **	−0.635 ***	0.994 ***	0.970 ***	0.977 ***
DTPA-Cd	−0.658 ***	−0.428 **	−0.642 ***		0.969 ***	0.971 ***
Shoot Cd conc.	−0.700 ***	−0.480 ***	−0.691 ***	0.969 ***		0.948 ***
Root Cd conc.	−0.628 ***	−0.407 **	−0.613 ***	0.971 ***	0.948 ***	

## Supplementary Materials

The following are available online at https://www.mdpi.com/2305-6304/8/2/36/s1. Table S1. BAI of Cd in maize plants exposed to MPs and Cd. Different letters following the mean values ± SD (*n* = 4) in the same column indicate significant differences using a one-way ANOVA followed by Duncan’s multiple range test (*p* < 0.05).
