# Supplementary material for: Effects of Co-Contamination of Microplastics and Cd on Plant Growth and Cd Accumulation
Source: Toxics. 2020 May 20;8(2):36. doi: 10.3390/toxics8020036 (PMC7356726; doi:10.3390/toxics8020036)
Supplement: Supplementary file 1 [file toxics-08-00036-s001.pdf]

Supplementary Material: Effects of Co-contamination of Microplastics and Cd on Plant Growth and Cd Accumulation

Fayuan Wang, Xiaoqing Zhang, Shuqi Zhang, Shuwu Zhang, Catharine A. Adams and Yuhuan Sun

Table 1. BAI of Cd in maize plants exposed to MPs and Cd. Different letters following the mean values ± SD ($n = 4$) in the same column indicate significant differences using a one-way ANOVA followed by Duncan's multiple range test ($p < 0.05$).

MPs treatment		BAI	
**Type**	**Dose**	**Shoots**	**Roots**
Control	0	3.91±0.47a	7.62±0.55ab
	0.1%	4.31±0.36a	8.95±1.26a
PE	1%	4.23±1.54a	6.66±0.30b
	10%	3.96±0.76a	8.61±0.43a
	0.1%	4.22±0.77a	8.69±1.68a
PS	1%	4.34±0.74a	8.15±1.45ab
	10%	3.73±0.24a	9.29±1.27a
